# From microRNA functions to microRNA therapeutics: Novel targets and novel drugs in breast cancer research and treatment

**DOI:** 10.3892/ijo.2013.2059

**Published:** 2013-08-12

**Authors:** ROBERTA PIVA, DEMETRIOS A. SPANDIDOS, ROBERTO GAMBARI

**Affiliations:** 1 Department of Biomedical and Specialty Surgical Sciences, Ferrara University, Ferrara, Italy ;; 2 Department of Clinical Virology, University of Crete School of Medicine, Heraklion, Crete, Greece ;; 3 Department of Life Sciences and Biotechnology, Ferrara University, Ferrara, Italy

**Keywords:** microRNAs, breast cancer, miR-221, Slug, p27 ^Kip1
^, peptide nucleic acid, microRNA replacement therapy, antagomiR

## Abstract

MicroRNAs (miRNAs or miRs) are a family of small non-coding RNAs that regulate gene expression by the sequence-selective targeting of mRNAs, leading to translational repression or mRNA degradation, depending on the degree of complementarity with target mRNA sequences. miRNAs play a crucial role in cancer. In the case of breast tumors, several studies have demonstrated a correlation between: i) the expression profile of oncogenic miRNAs (oncomiRs) or tumor suppressor miRNAs and ii) the tumorigenic potential of triple-negative [estrogen receptor (ER), progesterone receptor (PR) and Her2/neu] primary breast cancers. Among the miRNAs involved in breast cancer, miR-221 plays a crucial role for the following reasons: i) miR-221 is significantly overexpressed in triple-negative primary breast cancers; ii) the oncosuppressor p27 ^Kip1^ , a validated miR-221 target, is downregulated in aggressive cancer cell lines; and iii) the upregulation of a key transcription factor, Slug, appears to be crucial, since it binds to the miR-221/miR-222 promoter and is responsible for the high expression of the miR-221/miR-222 cluster in breast cancer cells. A Slug/miR-221 network has been suggested, linking miR-221 activity with the downregulation of a Slug repressor, leading to Slug/miR-221 upregulation and p27 ^Kip1^ downregulation. Interference with this process can be achieved using antisense miRNA (antagomiR) molecules targeting miR-221, inducing the down-regulation of Slug and the upregulation of p27 ^Kip1^ .

## Contents

1. Introduction
2. MicroRNAs and cancer
3. OncomiRs and MetastamiRs
4. Oncosuppressor microRNAs
5. MicroRNAs in breast tumors
6. Plasma miR-221 as a diagnostic marker in breast cancer
7. Connecting miRNA-221 with the expression of cellular genes altered in breast cancer cells: the Slug/miR-221 network
8. Effects of antagomiRs targeting oncomiRs
9. Novel drugs in miRNA therapeutics: peptide nucleic acid (PNA)
10. Conclusion

## 1. Introduction

**Figure 1.**
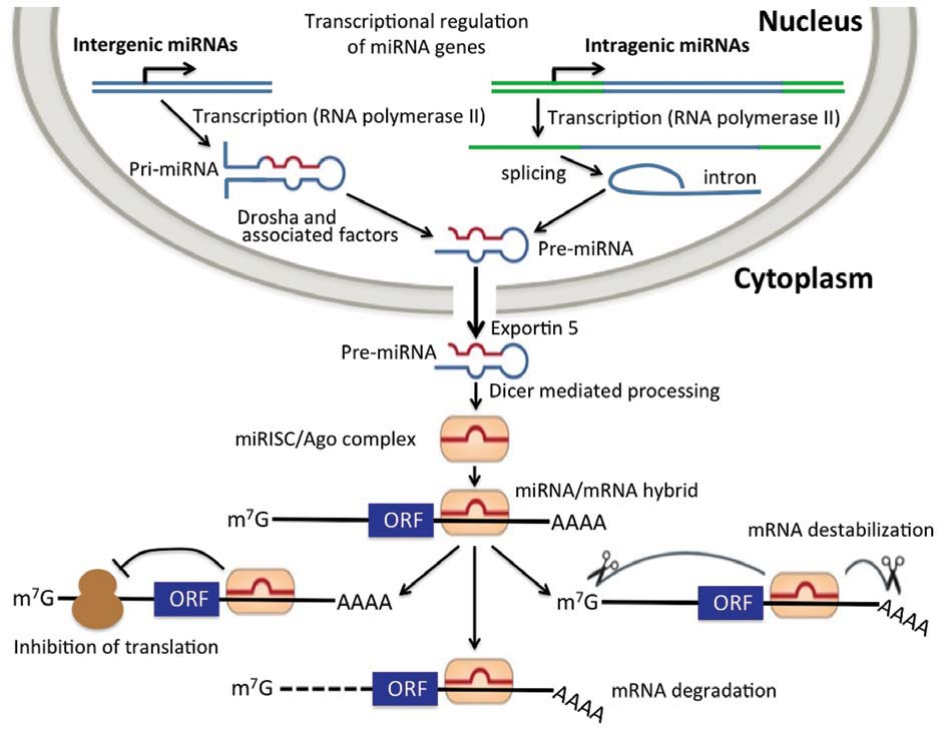
Biogenesis of microRNAs.

MicroRNAs (miRNAs or miRs) ( Fig. 1 ) are a family of small (19 to 25 nucleotides in length) non-coding RNAs that regulate gene expression by the sequence-selective targeting of mRNAs, leading to translational repression or mRNA degradation, depending on the degree of complementarity with target mRNA sequences ( 1 – 5 ) . Since their discovery and first characterization, the number of miRNA sequences deposited in the miRBase databases is increasing ( 6 – 10 ) . Considering that a single miRNA can target several mRNAs and a single mRNA may contain several signals for miRNA recognition in the 3′UTR sequence, it has been calculated that at least 10–40% of human mRNAs are a target for miRNAs ( 11 – 13 ) . Hence, the identification of validated targets of miRNAs is of great importance.

This specific field of miRNA research has confirmed that the complex networks constituted by miRNAs and mRNA targets coding for structural and regulatory proteins lead to the control of highly regulated biological functions, such as differentiation, cell cycle and apoptosis ( 14 – 16 ) . The low expression of a given miRNA is expected to be linked with a potential expression of target mRNAs. Conversely, the high expression of miRNAs is expected to negatively affect the biological functions of target mRNAs ( 1 – 5 ) .

Alterations in miRNA expression have been demonstrated to be associated with a variety of human pathologies, and the guided alterations of specific miRNAs have been suggested as novel approaches for the development of innovative therapeutic protocols. miRNA therapeutics is a novel field in which miRNA activity is the major target of intervention ( 17 – 21 ) . The inhibition of miRNA activity can be readily achieved by the use of small miRNA inhibitors, oligomers, including RNA, DNA and DNA analogues (miRNA antisense therapy) ( 19 , 22 – 28 ) . On the contrary, an increase in miRNA function (miRNA replacement therapy) can be achieved by the use of modified miRNA mimetics, such as plasmid or lentiviral vectors carrying miRNA sequences ( 20 , 21 , 29 – 37 ) .

## 2. MicroRNAs and cancer

miRNAs play a pivotal role in all the stages of cancer. The literature on this specific issue is impressive ( 22 – 37 ) . As a first example, miR-372 and miR-373 were identified as oncogenes, after a screening of hundreds of miRNAs in testicular germ cell tumors ( 38 ) . The mechanisms of action of these miRNAs involve the negative regulation of the expression of the LAST2 tumor suppressor gene, blocking the pathway of one of the key tumor suppressors, p53 ( 39 ) . Accordingly, using breast cancer MCF-7 cells as a model system, Huang *et al* demonstrated that miR-373 promotes tumor invasion and metastasis ( 40 ) . A similar tumor-promoting activity has been exhibited by miR-221 and miR-222, which can stimulate the proliferation of human prostate carcinoma cell lines following the inhibition of the expression of the tumor suppressor p27 ^Kip1^ ( 41 ) .

An opposite effect on tumor development has been displayed by other miRNAs; for instance miR-31 expression levels inversely correlate with the metastatic ability of breast tumor cell lines and the inhibition of miR-31 promotes metastasis. Another study revealed that miR-31 blocks several steps of metastasis, including local invasion, extravasation or initial survival at a distant site, and metastatic colonization ( 42 ) . Taken together, these data demonstrate that miRNAs play a double role in cancer, behaving both as oncogenes or tumor suppressor genes.

## 3. OncomiRs and metastamiRs

In general, a miRNA able to promote cancer targets mRNAs encoding tumor suppressor proteins, while miRNAs exhibiting tumor suppressor properties usually target mRNAs encoding oncoproteins. miRNAs which have been demonstrated to play a crucial role in the initiation and progression of human cancer are defined as oncogenic miRNAs (oncomiRs) ( 22 – 28 ) . Moreover, miRNAs have been firmly demonstrated to be involved in cancer metastasis (metastamiRs) ( 43 – 46 ) . Thus, therapeutic strategies involving miRNA silencing have been suggested, based on the roles of these small non-coding RNAs as oncogenes ( 22 – 28 ) .

Another very interesting feature of miRNAs has been found by studying cancer-associated miRNAs in different experimental model systems; cancer-specific miRNAs are present in extracellular body fluids and may play a crucial role in the cross-talk between cancer cells and surrounding normal cells ( 47 – 52 ) . Of note, evidence of the presence of miRNAs in serum, plasma and saliva supports their potential as an additional set of biomarkers for cancer. Extracellular miRNAs are protected by exosome-like structures, small intraluminal vesicles shed from a variety of cells (including cancer cells), with a biogenesis connected with the endosomal sorting complex required for transport machinery in multivesicular bodies. These extracellular structures, originally considered as a ‘garbage bag’ devoted to discarding degraded proteins, are now considered to play an important role as an intercellular communication tool. It is still unclear as to whether these exosome-associated miRNAs occur as a result of tumor cell death and lyses, or are actively excreted from tumor cells into the microenvironment. However, this novel secretory machinery of miRNAs may be involved in tumor-associated features, such as the enhancement of angiogenesis, the increase of cytokine secretion and migration to pre-metastatic niche. Table I illustrates a summarized list of oncomiRs and metastamiRs.

## 4. Oncosuppressor microRNAs

In addition to oncogenic activities, miRNAs exhibit, as has already been pointed out, oncosuppressor properties by targeting mRNAs encoding oncoproteins ( 29 – 37 ) . Piovan *et al* recently explored the interaction between certain miRNAs and transcriptional factors involved in determining cell fate, including the well known ‘genome guardian’, p53 ( 53 ) . They demonstrated that miR-205, an oncosuppressive miRNA lost in breast cancer, is directly transactivated by the oncosuppressor p53. Moreover, evaluating miR-205 expression in a panel of cell lines belonging to the highly aggressive triple-negative [estrogen receptor (ER), progesterone receptor (PR) and Her2/neu] breast cancer subtype, which still lacks an effective targeted therapy and is characterized by an extremely undifferentiated mesenchymal phenotype, the authors demonstrated that this miRNA is critically downregulated compared with a normal cell line. The re-expression of miR-205 strongly reduced cell proliferation, cell cycle progression and clonogenic potential *in vitro* , and inhibited tumor growth *in vivo* . The tumor suppressor activity of miR-205 is partially exerted by targeting of E2F1, one of the master regulators of cell cycle progression, and LAMC1, a component of the extracellular matrix involved in cell adhesion, proliferation and migration. In another study, Lee *et al* ( 54 ) , demonstrated that an estrogen-downregulated miRNA, miR-34b, acts as an oncosuppressor that targets cyclin D1 and Jagged-1 (JAG1) in an ERα-positive/wild-type p53 breast cancer cell line (MCF-7), as well as in ovarian and endometrial cells, but not in ERα-negative or mutant p53 breast cancer cell lines (T47D, MBA-MB-361 and MDA-MB-435). The negative association between ERα and miR-34b expression levels has also been found in ERα-positive breast cancer patients. In addition, the overexpression of miR-34b has been shown to inhibit ERα-positive breast tumor growth in an orthotopic mammary fat pad xenograft mouse model. Table II illustrates a summarized list of oncosuppressor miRNAs ( 29 – 37 , 55 – 58 ) .

## 5. MicroRNAs in breast tumors

As already presented in the previous chapters, miRNAs play a crucial role in breast tumors ( 59 – 73 ) . Several studies have been undertaken with the objective of determining the correlation between the expression profile of oncomiRs and tumor suppressor miRNAs, and, in particular, the tumorigenic potential of triple-negative primary breast cancers. In the study by Radojicic *et al* ( 68 ) 49 primary triple-negative breast cancer cases, along with 34 matched tumor-associated normal samples were investigated for the expression of 9 miRNAs using qRT-PCR. Correlations between the expression of miR-10b, miR-21, miR-122a, miR-145, miR-205, miR-210, miR-221, miR-222 and miR-296 and the pathological features of the tumors were examined, as well as the effects of miRNA expression on patient overall and cancer-specific survival. miR-21, miR-210 and miR-221 were significantly overexpressed, whereas miR-10b, miR-145, miR-205 and miR-122a were significantly underexpressed in the triple-negative primary breast cancers. Significant correlations among all the studied miRNAs were scored both in the breast cancer and control tissues. The expression of miR-222 and miR-296 did not exhibit any significant difference between the breast cancer and normal tissue.

A number of studies ( 61 – 67 , 74 ) have identified miR-221 and miR-222, as basal-like subtype-specific miRNAs that decrease the expression of epithelial-specific genes and increase the expression of mesenchymal-specific genes. In addition, the expression of these miRNAs increases cell migration and invasion; collectively, these are characteristics of epithelial-tomesenchymal transition (EMT). The basal-like transcription factor, FOSL1 (also known as Fra-1), directly stimulates the transcription of miR-221/222, and the abundance of these miRNAs decreases with the inhibition of MEK (mitogen-activated or extracellular signal-regulated protein kinase), placing miR-221/222 downstream of the RAS pathway. The miR-221/222-mediated reduction in E-cadherin abundance is dependent on their targeting of the 3′UTR of trichorhinophalangeal syndrome type 1 (TRPS1), which is a member of the GATA family of transcriptional repressors. TRPS1 inhibits EMT by directly repressing the expression of Zinc finger E-box-binding homeobox 2 (ZEB2). Therefore, these molecular data support the hypothesis that miR-221/222 contribute to the aggressive clinical behavior of basal-like breast cancer.

**Figure 2.**
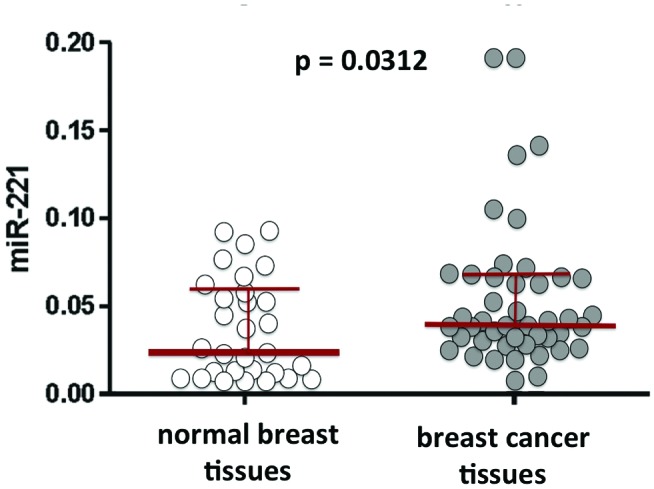
Distribution of the normalized expression levels of miR-221 in breast cancers and adjacent normal tissues. Bars indicate median values with inter-quartile range. Modified from the study by Radojicic *et al* ( 68 ).

Furthermore, the expression of miR-221 is clearly involved in chemoresistence. Studies have revealed an elevated expression of miR-221 in adriamycin-resistant MCF-7/ADR cells. In conclusion, several studies have indicated that miR-221 is one of the major miRNAs involved in breast cancer ( 75 ) . Representative results concerning miR-221 in breast cancer tissues are illustrated in Fig. 2 .

## 6. Plasma miR-221 as a diagnostic marker in breast cancer

In consideration of the importance of miR-221 in the tumor phenotype of breast cancer, several studies have been performed with the objective of analyzing miR-221 in biological fluids as a marker of breast cancer. Zhao *et al* ( 75 ) demonstrated that plasma miR-221 can be considered as a predictive biomarker for chemoresistance in breast cancer patients who have previously received neoadjuvant chemotherapy. The expression levels of circulating miR-221 were assessed in the plasma of 93 breast cancer patients who had previously received neoadjuvant chemotherapy (NAC), as well as in 32 healthy individuals. The correlation between miR-221 and clinicopathological features and chemosensitivity was also analyzed. The expression level of miR-221 was significantly associated with the hormone receptor (HR) status. Patients with higher plasma miR-221 levels tended to be HR-negative. Patients with varying miR-221 levels had significant differences in the overall response rate but not in the pathological complete response rate. These results indicate that plasma miR-221 may be a predictive biomarker for sensitivity to NAC in breast cancer patients.

## 7. Connecting miRNA-221 with the expression of cellular genes altered in breast cancer cells: the Slug/miR-221 network

**Figure 3.**
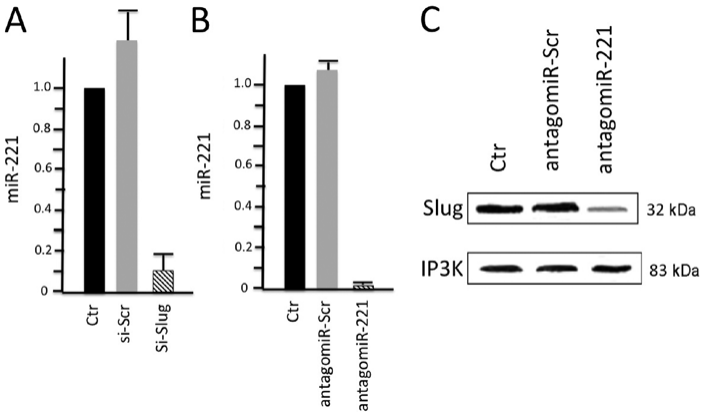
Effects of Slug silencing and antagomiR-221 treatment in MDA-MB-231 breast cancer cells. (A) Slug silencing, but not scrambled siRNA, markedly decreased miR-221 expression as demonstrated by qRT-PCR. (B and C) Treatment with antagomiR-221 abolished miR-221 expression and decreased Slug protein levels as shown by (B) qRT-PCR and (C) western blot analysis, respectively. IP3K was used as the loading control. Modified from the study by Lambertini *et al* ( 74 ).

This issue has great impact on the design of novel therapeutic approaches. On the one hand, it is very important to determine whether the expression of the miR-221/miR-222 cluster is under the transcriptional regulation of cellular proteins (for instance tumor-associated transcription factors). On the other hand, it is imperative to determine which mRNAs are specifically targeted by miR-221 (for instance tumor-suppressor mRNAs) determining the tumorigenic potential of this miRNA. Finally, it should be verified whether mRNAs regulated by miR-221 encode proteins able to regulate upstream miR-221 modifiers, therefore activating a ‘vicious intracellular cycle’. As regards these issues, a number of studies have been published. Lambertini *et al* ( 74 ) recently demonstrated that the Slug transcription factor binds to the miR-221/miR-222 promoter and is responsible for the high expression of the miR-221/miR-222 cluster in breast cancer cells. In order to investigate the possible correlation between the Slug transcription factor and miR-221, they performed Slug gene silencing in MDA-MB-231 breast cancer cells and evaluated the expression of genes involved in supporting the breast cancer phenotype by qRT-PCR and western blot analysis. Chromatin immunoprecipitation and wound healing assays were employed to determine a functional link between these two molecules. The results of their study ( Fig. 3 ) revealed that Slug silencing significantly decreased the level of miR-221 and vimentin, reactivated ERα and increased E-cadherin and TRPS1 expression ( 74 ) . It was demonstrated that miR-221 is a Slug target gene, and the authors identified a specific region of the miR-221 promoter that is transcriptionally active and binds the transcription factor Slug *in vivo* . In addition, they observed a more potent inhibiton of cell migration in the Slug-silenced cells, which retained residual miR-221 (approximately 38%), compared with antagomiR-221-treated cells with a complete knockdown of miR-221. As a whole, their study reported for the first time evidence of a correlation between the Slug transcription factor and miR-221 in breast cancer cells, suggesting that miR-221 expression is, at least in part, dependent on Slug, which is more effective than miR-221 in sustaining cell migration and invasion.

## 8. Effects of antagomiRs targeting oncomiRs

In miRNA therapeutics, by targeting oncomiRs and metastamiRs, several strategies have been performed to inhibit the functions of oncomiRs and metastamiRs. One of the most common approaches involves the use of antisense miRNAs (antagomiRs) capable of knocking down miRNAs. Velu *et al* ( 76 ) demonstrated the efficacy of the knockdown of miR-21, which is involved in myelopoiesis, using antagomiRs in primary murine bone marrow stem/progenitor cells. This approach has a clear potential impact in anticancer therapy, as demonstrated in a very recent study by Poltronieri *et al* ( 77 ) , who hypothesized that, as oncomiRs promote the growth of cancer cells and support survival during chemotherapy, thus miRNA-silencing therapies may be a valuable approach in conjunction with anticancer drugs and chemotherapy treatments. Specifically, they focused on miR-155, which they found overexpressed in different types of cancer. Of particular interest was the finding that GABA-A receptor downregulation was found to correlate with the glioma grade, with decreasing levels being associated with a higher grade of malignancy. The demonstration that the knockdown of miR-155 involves the re-expression of GABRA 1 protein *in vivo* has a great implication on the effectiveness of RNA-silencing approaches against miR-155, with the aim to control proliferation and signalling pathways regulated by the GABA-A receptor.

Another study also focused on potential anticancer therapy based on miRNA knockdown. Ma *et al* aimed to control mammary tumor metastasis ( 78 ) . They demonstrated that the systemic treatment of tumor-bearing mice with miR-10b antagomiRs suppresses breast cancer metastasis, both *in vitro* and *in vivo* . The silencing of miR-10b with antagomiRs significantly decreased miR-10b levels and increased the levels of a functionally important miR-10b target, Hoxd10. Of note, the administration of miR-10b antagomiRs to mice bearing highly metastatic cells did not reduce primary mammary tumor growth but markedly suppressed the formation of lung metastases in a sequence-specific manner. The miR-10b antagomiR, which is well tolerated by healthy animals, appears to be a promising candidate for the development of novel anti-metastatic agents.

## 9. Novel drugs in miRNA therapeutics: peptide nucleic acid (PNA)

**Figure 4.**
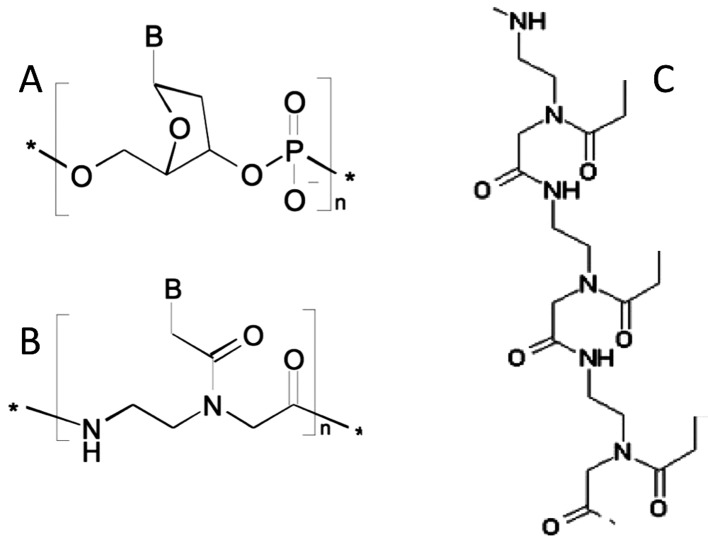
(A) DNA and (B) peptide nucleic acid (PNA) monomers. (C) PNA structure.

Peptide nucleic acid (PNA) ( Fig. 4 ) is a DNA analogue in which the sugar-phosphate backbone is replaced by N-(2-aminoethyl) glycine units ( 79 – 84 ) . These molecules efficiently hybridize with complementary DNA and RNA, forming a double helix with Watson-Crick base pairs ( 79 , 80 ) . Accordingly, PNA has been suggested for use in antisense and anti-gene therapy in a number of studies ( 83 – 86 ) . PNA is promising for RNA recognition, since it has a higher affinity for RNA than for DNA, is more specific, and is resistant to DNases and proteases ( 80 ) . PNA can be modified in order to achieve a better performance in terms of cellular permeation, higher affinity and specificity for the target DNA and RNA sequences ( 87 – 93 ) .

**Figure 5.**
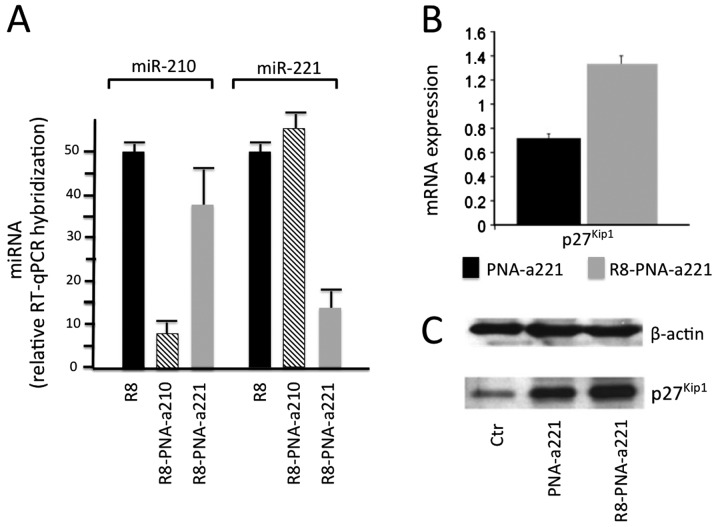
Effects of peptide nucleic acid (PNA)-based antagomiR against miR-221 in MDA-MB-231 breast cancer cells. (A) Effects of treatment of MDA-MB-231 cells (48 h) with 2 *μ* M PNA-a221, PNA-a210, R8-PNA-a221 and R8-PNA-a210 on hybridization to probes recognizing miR-210 and miR-221, as indicated. (B and C) Accumulation of p27 ^Kip1^ mRNA (B) in MDA-MB-231 cells treated for 96 h with 2 *μ* M PNA-a221 and Rpep-PNA-a221. (C) Western blot analysis performed on the same cellular population using antibody against p27 ^Kip1^ and against β-actin as reference protein. Modified from the study by Brognara *et al* ( 94 ).

In the case of the development of PNA-based miRNA therapeutics for altering gene expression in breast cancer cells, PNA targeting miR-221 has shown to specifically interact with miR-221 expressed in aggressive breast cancer cell lines ( 94 ) . In order to maximize uptake in target cells, a polyarginine-peptide (R8) was conjugated, generating an anti-miR-221 PNA (R8-PNA-a221) displaying very high affinity for RNA and efficient uptake within target cells without the need of transfection reagents. Unmodified PNA with the same sequence displayed RNA binding, but cellular uptake was very poor. Consistently, only R8-PNA-a221 markedly inhibited miR-221 in MDA-MB-231 breast cancer cells. This is illustrated in Fig. 5A , describing the effects of two PNA-based antagomiRs, R8-PNA-a210 and R8-PNAa221, targeting miR-210 and miR-221, respectively. As it is clearly evident, R8-PNA-a210 inhibits miR-210 but not miR-221 and vice-versa, R8-PNAa221 inhibits miR-221 but not miR-210. Therefore, targeting miR-221 with R8-PNAa221 resulted in i) a specific decrease in the hybridization levels of miR-221 measured by qRT-PCR; and ii) the upregulation of p27 ^Kip1^ , mRNA and protein, measured by qRT-PCR and western blot analysis ( Fig. 5B and C ).

While research on anti-miR PNA has just begun ( 95 – 100 ) , pre-clinical results are expected in the near future to sustain the hypothesis that miRNA-targeted molecules based on PNA can be successfully applied to treat human diseases. An example was recently reported by Yan *et al* ( 101 ) , who addressed the potential effects of PNA-anti-miR-21 *in vivo* on the growth of breast cancer cells. In their experiments, MCF-7 cells treated with PNA-anti-miR-21 or PNA-control were subcutaneously injected into female nude mice. Detectable tumor masses were observed in only 5/8 of mice in the MCF/PNA-anti-miR-21 group, while much larger tumors were detected in all mice in the MCF/PNA-control group. Both the tumor weight and number showed that MCF/PNA-control cells formed larger tumors more rapidly than the MCF/PNA-anti-miR-21 cells in nude mice.

## 10. Conclusion

**Figure 6.**
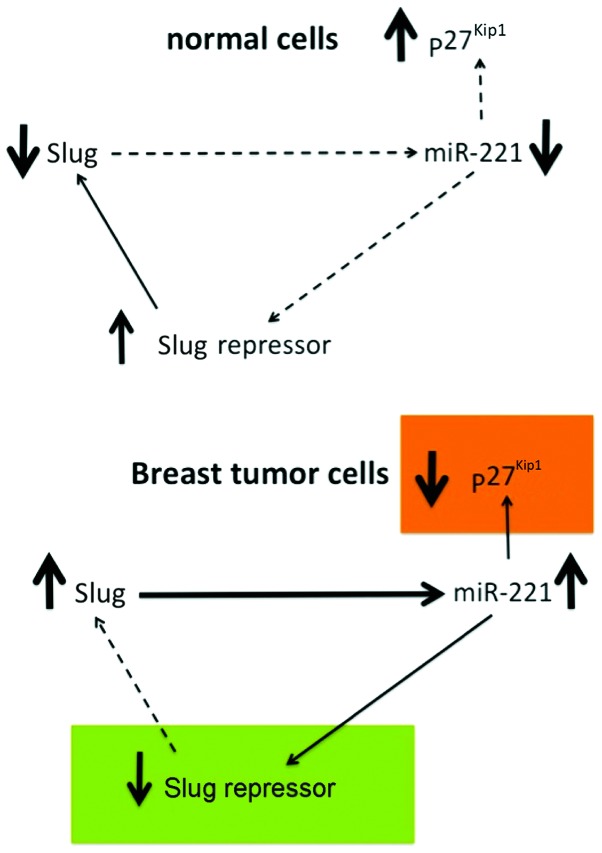
Schematic diagram outlining the interactions between miR-221, Slug and p27 ^Kip1^ in breast cancer.

The large number of studies on miRNAs in different types of *in vitro* and *in vivo* experimental models, both in basic and applied research, demonstrate the high prognostic, diagnostic and therapeutic value of these recently discovered molecules. The demonstration of the functions of these molecules has not only led to the discovery of a new system of regulation of gene expression that renews the concepts of molecular biology known to date, but has also allowed the development of novel clinical tools. The close collaboration between researchers and clinicians will be required in the near future to enhance the knowledge on the biology of miRNAs and exploit their potential to improve human health. In this context, miR-221 plays a crucial role in breast cancer for the following reasons: i) miR-221 is significantly overexpressed in triple-negative primary breast cancers; ii) the oncosuppressor p27 ^Kip1^ , a validated target of miR-221, is downregulated in aggressive cancer cell lines; and iii) the upregulation of a key transcription factor, Slug, appears to be crucial, since it binds to the miR-221/miR-222 promoter and is responsible for the high expression of the miR-221/miR-222 cluster in breast cancer cells. A Slug/miR-221 network is thus proposed, linking miR-221 activity with the downregulation of a Slug repressor, leading to Slug/miR-221 upregulation and p27 ^Kip1^ downregulation ( Fig. 6 ). Interference with this process can be achieved using antagomiR molecules targeting miR-221, inducing the downregulation of Slug and the upregulation of p27 ^Kip1^ . Targeting miR-221 with DNA analogues may be clinically relevant in antagomiR therapeutics.

## Figures and Tables

**Table I.** Examples of oncomiRs suitable for antagomiR-based miRNA targeted therapy of cancer.

Cells/tissues	miRNA target	Modulated mRNA	Effects following antagomiR treatement	Authors/(Refs.)
Human glioblastoma	miR-27a	FOXO3a	Suppression of U87 growth * in vitro * and * in vivo *	Ge * et al * ( 22 )
Cutaneous squamous cell carcinoma (SCC)	miR-155	CDC73	Decreased cell viability, increased apoptosis and marked regression of xenografts in nude mice	Rather * et al * ( 23 )
Malignant astrocytoma cells	miR-335	Daam1	Growth arrest, cell apoptosis, invasion repression and marked regression of astrocytoma xenografts	Shu * et al * ( 24 )
Neuroblastoma	miR-92	DKK3	Increased release of the tumor suppressor Dickkopf-3 (DKK3), a secreted protein of the DKK family of Wnt regulators	Haug * et al * ( 25 )
Glioma	miR-381	LRRC4	Decreased cell proliferation and tumor growth	Tang * et al * ( 26 )
Breast cancer	miR-10b	Hoxd10	Suppression of formation of lung metastases	Ma * et al * ( 27 )
Prostate cancer	miR-221/miR-222	p27	Reduction of tumor growth	Mercatelli * et al * ( 28 )

**Table II.** miRNAs acting as tumor suppressor genes and are suitable for replacement therapy of cancer: selected examples.

Tumor type	miRNA	Modulated mRNA	Effects following pre-miRNA administration	Authors/(Refs.)
Acute leukemia	miR-27a	Bax and Bad	Inhibition of cell growth due, at least in part, to increased cellular apoptosis	Scheibner * et al * ( 29 )
Oral squamous cell carcinoma (OSCC)	miR-596	LGALS3BP	Growth inhibition	Endo * et al * ( 30 )
Breast cancer	miR-302	AKT1 and RAD52	Sensitized radioresistant breast cancer cells to ionizing radiation	Liang * et al * ( 31 )
Chronic myelogenous leukemia (CML) cells	miR-33a	Pim-1	Decelerated cell proliferation	Thomas * et al * ( 32 )
Colon carcinoma	miR-33a	Pim-1	Reduced tumor proliferation	Ibrahim * et al * ( 33 )
Colon carcinoma	miR-145	c-Myc and ERK5	Reduced tumor proliferation and increased apoptosis	Ibrahim * et al * ( 33 )
Lung cancer	miR-34a	Repression of c-Met, Bcl-2; partial repression of CDK4	Block of tumor growth	Wiggins * et al * ( 34 )
Lung cancer	miR-let7	Negative regulation of the cell cycle oncogenes RAS, MYC and HMGA2	Reduction of tumor growth	Trang * et al * ( 35 )
Non-small cell lung adenocarcinomas, A549 cells	miR-29b	CDK6, DNMT3B, MCL-1	Inhibition of tumorigenicity * in vivo *	Wu * et al * ( 36 )
Acute myeloid leukemia (AML)	miR-29b	Downregulation of DNMTs, CDK6, SP1, KIT and FLT3	Decreased AML cell growth and impairement of colony formation; longer survival of treated mice; improvement of anti-leukemic activity of decitabine	Huang * et al * ( 37 )

## Abbreviations:

miRNA: microRNA;
ER: estrogen receptor;
PNA: peptide nucleic acid;
3′UTR: 3′ untranslated region

## References

[1] 
Filipowicz
 
W
, 
Jaskiewicz
 
L
, 
Kolb
 
FA
, 
Pillai
 
RS
 (2005). Post-transcriptional gene silencing by siRNAs and miRNAs. Curr Opin Struc Biol.

[2] 
He
 
L
, 
Hannon
 
GJ
 (2004). MicroRNAs: small RNAs with a big role in gene regulation. Nat Rev Genet.

[3] 
Kozomara
 
A
, 
Griffiths-Jones
 
S
 (2011). miRBase: integrating microRNA annotation and deep-sequencing data. Nucleic Acids Res.

[4] 
Krol
 
J
, 
Loedige
 
I
, 
Filipowicz
 
W
 (2010). The widespread regulation of microRNA biogenesis, function and decay. Nat Rev Genet.

[5] 
Sontheimer
 
EJ
, 
Carthew
 
RW
 (2005). Silence from within: endogenous siRNAs and miRNAs. Cell.

[6] 
Taccioli
 
C
, 
Fabbri
 
E
, 
Visone
 
R
, 
Volinia
 
S
, 
Calin
 
GA
, 
Fong
 
LY
 (2009). UCbase and miRfunc: a database of ultracon-served sequences and microRNA function. Nucleic Acids Res.

[7] 
Griffiths-Jones
 
S
 (2006). miRBase: the microRNA sequence database. Methods Mol Biol.

[8] 
Witwer
 
KW
 (2013). Data submission and quality in microarray-based microRNA profiling. Clin Chem.

[9] 
Sablok
 
G
, 
Milev
 
I
, 
Minkov
 
G
, 
Minkov
 
I
, 
Varotto
 
C
, 
Yahubyan
 
G
, 
Baev
 
V
 (
2013
). isomiRex: Web-based identification of microRNAs, isomiR variations and differential expression using next-generation sequencing datasets. FEBS Lett.

[10] 
Russo
 
F
, 
Di Bella
 
S
, 
Nigita
 
G
, 
Macca
 
V
, 
Laganà
 
A
, 
Giugno
 
R
, 
Pulvirenti
 
A
, 
Ferro
 
A
 (2012). miRandola: extracellular circulating microRNAs database. PLoS One.

[11] 
Krützfeldt
 
J
, 
Kuwajima
 
S
, 
Braich
 
R
, 
Rajeev
 
KG
, 
Pena
 
J
, 
Tuschl
 
T
, 
Manoharan
 
M
, 
Stoffel
 
M
 (2007). Specificity, duplex degradation and subcellular localization of antagomirs. Nucleic Acids Res.

[12] 
Dalmay
 
T
 (2013). Mechanism of miRNA-mediated repression of mRNA translation. Essays Biochem.

[13] 
Jiang
 
Q
, 
Wang
 
Y
, 
Hao
 
Y
, 
Juan
 
L
, 
Teng
 
M
, 
Zhang
 
X
, 
Li
 
M
, 
Wang
 
G
, 
Liu
 
Y
 (2009). miR2Disease: a manually curated database for microRNA deregulation in human disease. Nucleic Acids Res.

[14] 
Subramanian
 
S
, 
Steer
 
CJ
 (2010). MicroRNAs as gatekeepers of apoptosis. J Cell Physiology.

[15] 
Wang
 
YM
, 
Blelloch
 
R
 (2010). Cell cycle regulation by MicroRNAs in embryonic stem cells. Cancer Res.

[16] 
Alvarez-Garcia
 
I
, 
Miska
 
EA
 (2005). MicroRNA functions in animal development and human disease. Development.

[17] 
Tsai
 
LM
, 
Yu
 
D
 (2010). MicroRNAs in common diseases and potential therapeutic applications. Clin Exp Pharmacol Physiol.

[18] 
Hemida
 
MG
, 
Ye
 
X
, 
Thair
 
S
, 
Yang
 
D
 (2010). Exploiting the therapeutic potential of microRNAs in viral diseases: expectations and limitations. Mol Diagn Ther.

[19] 
Kota
 
SK
, 
Balasubramanian
 
S
 (2010). Cancer therapy via modulation of micro RNA levels: a promising future. Drug Discov Today.

[20] 
Bader
 
AG
, 
Brown
 
D
, 
Winkler
 
M
 (2010). The promise of microRNA replacement therapy. Cancer Res.

[21] 
Sibley
 
CR
, 
Seow
 
Y
, 
Wood
 
MJ
 (2010). Novel RNA-based strategies for therapeutic gene silencing. Mol Ther.

[22] 
Ge
 
YF
, 
Sun
 
J
, 
Jin
 
CJ
, 
Cao
 
BQ
, 
Jiang
 
ZF
, 
Shao
 
JF
 (2013). AntagomiR-27a targets FOXO3a in glioblastoma and suppresses U87 cell growth in vitro and in vivo. Asian Pac J Cancer Prev.

[23] 
Rather
 
MI
, 
Nagashri
 
MN
, 
Swamy
 
SS
, 
Gopinath
 
KS
, 
Kumar
 
A
 (2013). Oncogenic microRNA-down-regulates tumor suppressor CDC73 and promotes oral squamous cell carcinoma cell proliferation: implications for cancer therapeutics. J Biol Chem.

[24] 
Shu
 
M
, 
Zheng
 
X
, 
Wu
 
S
, 
Lu
 
H
, 
Leng
 
T
, 
Zhu
 
W
, 
Zhou
 
Y
, 
Ou
 
Y
, 
Lin
 
X
, 
Lin
 
Y
, 
Xu
 
D
, 
Zhou
 
Y
, 
Yan
 
G
 (
2011
). Targeting oncogenic miR-335 inhibits growth and invasion of malignant astrocytoma cells. Mol Cancer.

[25] 
Haug
 
BH
, 
Henriksen
 
JR
, 
Buechner
 
J
, 
Geerts
 
D
, 
Tømte
 
E
, 
Kogner
 
P
, 
Martinsson
 
T
, 
Flægstad
 
T
, 
Sveinbjørnsson
 
B
, 
Einvik
 
C
 (2011). MYCN-regulated miRNA-92 inhibits secretion of the tumor suppressor DICKKOPF-3 (DKK3) in neuroblastoma. Carcinogenesis.

[26] 
Tang
 
H
, 
Liu
 
X
, 
Wang
 
Z
, 
She
 
X
, 
Zeng
 
X
, 
Deng
 
M
, 
Liao
 
Q
, 
Guo
 
X
, 
Wang
 
R
, 
Li
 
X
, 
Zeng
 
F
, 
Wu
 
M
, 
Li
 
G
 (2011). Interaction of hsa-miR-381 and glioma suppressor LRRC4 is involved in glioma growth. Brain Res.

[27] 
Ma
 
L
, 
Reinhardt
 
F
, 
Pan
 
E
, 
Soutschek
 
J
, 
Bhat
 
B
, 
Marcusson
 
EG
, 
Teruya-Feldstein
 
J
, 
Bell
 
GW
, 
Weinberg
 
RA
 (2010). Therapeutic silencing of miR-10b inhibits metastasis in a mouse mammary tumor model. Nat Biotechnol.

[28] 
Mercatelli
 
N
, 
Coppola
 
V
, 
Bonci
 
D
, 
Miele
 
F
, 
Costantini
 
A
, 
Guadagnoli
 
M
, 
Bonanno
 
E
, 
Muto
 
G
, 
Frajese
 
GV
, 
De Maria
 
R
, 
Spagnoli
 
LG
, 
Farace
 
MG
, 
Ciafrè
 
SA
 (2008). The inhibition of the highly expressed miR-221 and miR-222 impairs the growth of prostate carcinoma xenografts in mice. PLoS One.

[29] 
Scheibner
 
KA
, 
Teaboldt
 
B
, 
Hauer
 
MC
, 
Chen
 
X
, 
Cherukuri
 
S
, 
Guo
 
Y
, 
Kelley
 
SM
, 
Liu
 
Z
, 
Baer
 
MR
, 
Heimfeld
 
S
, 
Civin
 
CI
 (2012). MiR-27a functions as a tumor suppressor in acute leukemia by regulating 14-3-3θ. PLoS One.

[30] 
Endo
 
H
, 
Muramatsu
 
T
, 
Furuta
 
M
, 
Uzawa
 
N
, 
Pimkhaokham
 
A
, 
Amagasa
 
T
, 
Inazawa
 
J
, 
Kozaki
 
K
 (2013). Potential of tumor-suppressive miR-596 targeting LGALS3BP as a therapeutic agent in oral cancer. Carcinogenesis.

[31] 
Liang
 
Z
, 
Ahn
 
J
, 
Guo
 
D
, 
Votaw
 
JR
, 
Shim
 
H
 (2013). MicroRNA-302 replacement therapy sensitizes breast cancer cells to ionizing radiation. Pharm Res.

[32] 
Thomas
 
M
, 
Lange-Grünweller
 
K
, 
Weirauch
 
U
, 
Gutsch
 
D
, 
Aigner
 
A
, 
Grünweller
 
A
, 
Hartmann
 
RK
 (2012). The proto-oncogene Pim-1 is a target of miR-33a. Oncogene.

[33] 
Ibrahim
 
AF
, 
Weirauch
 
U
, 
Thomas
 
M
, 
Grünweller
 
A
, 
Hartmann
 
RK
, 
Aigner
 
A
 (2011). MicroRNA replacement therapy for miR-145 and miR-33a is efficacious in a model of colon carcinoma. Cancer Res.

[34] 
Wiggins
 
JF
, 
Ruffino
 
L
, 
Kelnar
 
K
, 
Omotola
 
M
, 
Patrawala
 
L
, 
Brown
 
D
, 
Bader
 
AG
 (2010). Development of a lung cancer therapeutic based on the tumor suppressor microRNA-34. Cancer Res.

[35] 
Trang
 
P
, 
Wiggins
 
JF
, 
Daige
 
CL
, 
Cho
 
C
, 
Omotola
 
M
, 
Brown
 
D
, 
Weidhaas
 
JB
, 
Bader
 
AG
, 
Slack
 
FJ
 (2011). Systemic delivery of tumor suppressor microRNA mimics using a neutral lipid emulsion inhibits lung tumors in mice. Mol Ther.

[36] 
Wu
 
Y
, 
Crawford
 
M
, 
Mao
 
Y
, 
Lee
 
RJ
, 
Davis
 
IC
, 
Elton
 
TS
, 
Lee
 
LJ
, 
Nana-Sinkam
 
SP
 (2013). Therapeutic delivery of microRNA-29b by cationic lipoplexes for lung cancer. Mol Ther Nucleic Acids.

[37] 
Huang
 
X
, 
Schwind
 
S
, 
Yu
 
B
, 
Santhanam
 
R
, 
Wang
 
H
, 
Hoellerbauer
 
P
, 
Mims
 
A
, 
Klisovic
 
R
, 
Walker
 
AR
, 
Chan
 
KK
, 
Blum
 
W
, 
Perrotti
 
D
, 
Byrd
 
JC
, 
Bloomfield
 
CD
, 
Caligiuri
 
MA
, 
Lee
 
RJ
, 
Garzon
 
R
, 
Muthusamy
 
N
, 
Lee
 
LJ
, 
Marcucci
 
G
 (2013). Targeted delivery of microRNA-29b by transferrin-conjugated anionic lipopolyplex nanoparticles: a novel therapeutic strategy in acute myeloid leukemia. Clin Cancer Res.

[38] 
Voorhoeve
 
PM
, 
le Sage
 
C
, 
Schrier
 
M
, 
Gillis
 
AJ
, 
Stoop
 
H
, 
Nagel
 
R
, 
Liu
 
YP
, 
van Duijse
 
J
, 
Drost
 
J
, 
Griekspoor
 
A
, 
Zlotorynski
 
E
, 
Yabuta
 
N
, 
De Vita
 
G
, 
Nojima
 
H
, 
Looijenga
 
LH
, 
Agami
 
R
 (2006). A genetic screen implicates miRNA-372 and miRNA-373 as oncogenes in testicular germ cell tumors. Cell.

[39] 
Voorhoeve
 
PM
, 
le Sage
 
C
, 
Schrier
 
M
, 
Gillis
 
AJ
, 
Stoop
 
H
, 
Nagel
 
R
, 
Liu
 
YP
, 
van Duijse
 
J
, 
Drost
 
J
, 
Griekspoor
 
A
, 
Zlotorynski
 
E
, 
Yabuta
 
N
, 
De Vita
 
G
, 
Nojima
 
H
, 
Looijenga
 
LH
, 
Agami
 
R
 (2007). A genetic screen implicates miRNA-372 and miRNA-373 as oncogenes in testicular germ cell tumors. Adv Exp Med Biol.

[40] 
Huang
 
Q
, 
Gumireddy
 
K
, 
Schrier
 
M
, 
le Sage
 
C
, 
Nagel
 
R
, 
Nair
 
S
, 
Egan
 
DA
, 
Li
 
A
, 
Huang
 
G
, 
Klein-Szanto
 
AJ
, 
Gimotty
 
PA
, 
Katsaros
 
D
, 
Coukos
 
G
, 
Zhang
 
L
, 
Puré
 
E
, 
Agami
 
R
 (2008). The microRNAs miR-373 and miR-520c promote tumour invasion and metastasis. Nat Cell Biol.

[41] 
Galardi
 
S
, 
Mercatelli
 
N
, 
Giorda
 
E
, 
Massalini
 
S
, 
Frajese
 
GV
, 
Ciafrè
 
SA
, 
Farace
 
MG
 (2007). miR-221 and miR-222 expression affects the proliferation potential of human prostate carcinoma cell lines by targeting p27
^
Kip1
^. J Biol Chem.

[42] 
Valastyan
 
S
, 
Reinhardt
 
F
, 
Benaich
 
N
, 
Calogrias
 
D
, 
Szász
 
AM
, 
Wang
 
ZC
, 
Brock
 
JE
, 
Richardson
 
AL
, 
Weinberg
 
RA
 (2009). A pleiotropically acting microRNA, miR-31, inhibits breast cancer metastasis. Cell.

[43] 
Hurst
 
DR
, 
Edmonds
 
MD
, 
Welch
 
DR
 (2009). Metastamir: the field of metastasis-regulatory microRNA is spreading. Cancer Res.

[44] 
Wotschofsky
 
Z
, 
Liep
 
J
, 
Meyer
 
HA
, 
Jung
 
M
, 
Wagner
 
I
, 
Disch
 
AC
, 
Schaser
 
KD
, 
Melcher
 
I
, 
Kilic
 
E
, 
Busch
 
J
, 
Weikert
 
S
, 
Miller
 
K
, 
Erbersdobler
 
A
, 
Mollenkopf
 
HJ
, 
Jung
 
K
 (2012). Identification of metastamirs as metastasis-associated microRNAs in clear cell renal cell carcinomas. Int J Biol Sci.

[45] 
Taylor
 
MA
, 
Sossey-Alaoui
 
K
, 
Thompson
 
CL
, 
Danielpour
 
D
, 
Schiemann
 
WP
 (2013). TGF-β upregulates miR-181a expression to promote breast cancer metastasis. J Clin Invest.

[46] 
Welch
 
DR
, 
Hurst
 
DR
 (
2013
). Unraveling the ‘TGF-β paradox’ one metastamir at a time. Breast Cancer Res.

[47] 
Moldovan
 
L
, 
Batte
 
K
, 
Wang
 
Y
, 
Wisler
 
J
, 
Piper
 
M
 (2013). Analyzing the circulating microRNAs in exosomes/extracellular vesicles from serum or plasma by qRT-PCR. Methods Mol Biol.

[48] 
Chen
 
X
, 
Liang
 
H
, 
Zhang
 
J
, 
Zen
 
K
, 
Zhang
 
CY
 (2012). Horizontal transfer of microRNAs: molecular mechanisms and clinical applications. Protein Cell.

[49] 
Kosaka
 
N
, 
Ochiya
 
T
 (
2011
). Unraveling the mystery of cancer by secretory microRNA: horizontal microRNA transfer between living cells. Front Genet.

[50] 
Chen
 
X
, 
Liang
 
H
, 
Zhang
 
J
, 
Zen
 
K
, 
Zhang
 
CY
 (2012). Secreted microRNAs: a new form of intercellular communication. Trends Cell Biol.

[51] 
Ramachandran
 
S
, 
Palanisamy
 
V
 (2012). Horizontal transfer of RNAs: exosomes as mediators of intercellular communication. Wiley Interdiscip Rev RNA.

[52] 
Muralidharan-Chari
 
V
, 
Clancy
 
JW
, 
Sedgwick
 
A
, 
D’Souza-Schorey
 
C
 (2010). Microvesicles: mediators of extracellular communication during cancer progression. J Cell Sci1.

[53] 
Piovan
 
C
, 
Palmieri
 
D
, 
Di Leva
 
G
, 
Braccioli
 
L
, 
Casalini
 
P
, 
Nuovo
 
G
, 
Tortoreto
 
M
, 
Sasso
 
M
, 
Plantamura
 
I
, 
Triulzi
 
T
, 
Taccioli
 
C
, 
Tagliabue
 
E
, 
Iorio
 
MV
, 
Croce
 
CM
 (2012). Oncosuppressive role of p53-induced miR-205 in triple negative breast cancer. Mol Oncol.

[54] 
Lee
 
YM
, 
Lee
 
JY
, 
Ho
 
CC
, 
Hong
 
QS
, 
Yu
 
SL
, 
Tzeng
 
CR
, 
Yang
 
PC
, 
Chen
 
HW
 (2011). miRNA-34b as a tumor suppressor in estrogen-dependent growth of breast cancer cells. Breast Cancer Res.

[55] 
Iorio
 
MV
, 
Croce
 
CM
 (2012). Causes and consequences of microRNA dysregulation. Cancer J.

[56] 
Xu
 
X
, 
Chen
 
H
, 
Lin
 
Y
, 
Hu
 
Z
, 
Mao
 
Y
, 
Wu
 
J
, 
Xu
 
X
, 
Zhu
 
Y
, 
Li
 
S
, 
Zheng
 
X
, 
Xie
 
L
 (2013). MicroRNA-409-3p inhibits migration and invasion of bladder cancer cells via targeting c-Met. Mol Cells.

[57] 
He
 
J
, 
Deng
 
Y
, 
Yang
 
G
, 
Xie
 
W
 (2013). MicroRNA-203 down-regulation is associated with unfavorable prognosis in human glioma. J Surg Oncol.

[58] 
Iorio
 
MV
, 
Croce
 
CM
 (2009). MicroRNAs in cancer: small molecules with a huge impact. J Clin Oncol.

[59] 
Volinia
 
S
, 
Galasso
 
M
, 
Sana
 
ME
, 
Wise
 
TF
, 
Palatini
 
J
, 
Huebner
 
K
, 
Croce
 
CM
 (2012). Breast cancer signatures for invasiveness and prognosis defined by deep sequencing of microRNA. Proc Natl Acad Sci USA.

[60] 
Lu
 
Y
, 
Roy
 
S
, 
Nuovo
 
G
, 
Ramaswamy
 
B
, 
Miller
 
T
, 
Shapiro
 
C
, 
Jacob
 
ST
, 
Majumder
 
S
 (2011). Anti-microRNA-222 (anti-miR-222) and -181B suppress growth of tamoxifen-resistant xenografts in mouse by targeting TIMP3 protein and modulating mitogenic signal. J Biol Chem.

[61] 
Shah
 
MY
, 
Calin
 
GA
 (
2011
). MicroRNAs miR-221 and miR-222: a new level of regulation in aggressive breast cancer. Genome Med.

[62] 
Stinson
 
S
, 
Lackner
 
MR
, 
Adai
 
AT
, 
Yu
 
N
, 
Kim
 
HJ
, 
O’Brien
 
C
, 
Spoerke
 
J
, 
Jhunjhunwala
 
S
, 
Boyd
 
Z
, 
Januario
 
T
, 
Newman
 
RJ
, 
Yue
 
P
, 
Bourgon
 
R
, 
Modrusan
 
Z
, 
Stern
 
HM
, 
Warming
 
S
, 
de Sauvage
 
FJ
, 
Amler
 
L
, 
Yeh
 
RF
, 
Dornan
 
D
 (2011). miR-221/222 targeting of trichorhinophalangeal 1 (TRPS1) promotes epithelial-to-mesenchymal transition in breast cancer. Sci Signal.

[63] 
Cochrane
 
DR
, 
Cittelly
 
DM
, 
Howe
 
EN
, 
Spoelstra
 
NS
, 
McKinsey
 
EL
, 
LaPara
 
K
, 
Elias
 
A
, 
Yee
 
D
, 
Richer
 
JK
 (2010). MicroRNAs link estrogen receptor alpha status and Dicer levels in breast cancer. Horm Cancer.

[64] 
Yoshimoto
 
N
, 
Toyama
 
T
, 
Takahashi
 
S
, 
Sugiura
 
H
, 
Endo
 
Y
, 
Iwasa
 
M
, 
Fujii
 
Y
, 
Yamashita
 
H
 (2011). Distinct expressions of microRNAs that directly target estrogen receptor α in human breast cancer. Breast Cancer Res Treat.

[65] 
Stinson
 
S
, 
Lackner
 
MR
, 
Adai
 
AT
, 
Yu
 
N
, 
Kim
 
HJ
, 
O’Brien
 
C
, 
Spoerke
 
J
, 
Jhunjhunwala
 
S
, 
Boyd
 
Z
, 
Januario
 
T
, 
Newman
 
RJ
, 
Yue
 
P
, 
Bourgon
 
R
, 
Modrusan
 
Z
, 
Stern
 
HM
, 
Warming
 
S
, 
de Sauvage
 
FJ
, 
Amler
 
L
, 
Yeh
 
RF
, 
Dornan
 
D
 (2011). TRPS1 targeting by miR-221/222 promotes the epithelial-to-mesenchymal transition in breast cancer. Sci Signal.

[66] 
Guttilla
 
IK
, 
Phoenix
 
KN
, 
Hong
 
X
, 
Tirnauer
 
JS
, 
Claffey
 
KP
, 
White
 
BA
 (2012). Prolonged mammosphere culture of MCF-7 cells induces an EMT and repression of the estrogen receptor by microRNAs. Breast Cancer Res Treat.

[67] 
Gordanpour
 
A
, 
Stanimirovic
 
A
, 
Nam
 
RK
, 
Moreno
 
CS
, 
Sherman
 
C
, 
Sugar
 
L
, 
Seth
 
A
 (2011). miR-221 is down-regulated in TMPRSS2: ERG fusion-positive prostate cancer. Anticancer Res.

[68] 
Radojicic
 
J
, 
Zaravinos
 
A
, 
Vrekoussis
 
T
, 
Kafousi
 
M
, 
Spandidos
 
DA
, 
Stathopoulos
 
EN
 (2011). MicroRNA expression analysis in triple-negative (ER, PR and Her2/neu) breast cancer. Cell Cycle.

[69] 
Pelletier
 
C
, 
Speed
 
WC
, 
Paranjape
 
T
, 
Keane
 
K
, 
Blitzblau
 
R
, 
Hollestelle
 
A
, 
Safavi
 
K
, 
van den Ouweland
 
A
, 
Zelterman
 
D
, 
Slack
 
FJ
, 
Kidd
 
KK
, 
Weidhaas
 
JB
 (2011). Rare BRCA1 haplotypes including 3’UTR SNPs associated with breast cancer risk. Cell Cycle.

[70] 
Rao
 
X
, 
Di Leva
 
G
, 
Li
 
M
, 
Fang
 
F
, 
Devlin
 
C
, 
Hartman-Frey
 
C
, 
Burow
 
ME
, 
Ivan
 
M
, 
Croce
 
CM
, 
Nephew
 
KP
 (2011). MicroRNA-221/222 confers breast cancer fulvestrant resistance by regulating multiple signaling pathways. Oncogene.

[71] 
Zhou
 
M
, 
Liu
 
Z
, 
Zhao
 
Y
, 
Ding
 
Y
, 
Liu
 
H
, 
Xi
 
Y
, 
Xiong
 
W
, 
Li
 
G
, 
Lu
 
J
, 
Fodstad
 
O
, 
Riker
 
AI
, 
Tan
 
M
 (2010). MicroRNA-125b confers the resistance of breast cancer cells to paclitaxel through suppression of pro-apoptotic Bcl-2 antagonist killer 1 (Bak1) expression. J Biol Chem.

[72] 
Di Leva
 
G
, 
Gasparini
 
P
, 
Piovan
 
C
, 
Ngankeu
 
A
, 
Garofalo
 
M
, 
Taccioli
 
C
, 
Iorio
 
MV
, 
Li
 
M
, 
Volinia
 
S
, 
Alder
 
H
, 
Nakamura
 
T
, 
Nuovo
 
G
, 
Liu
 
Y
, 
Nephew
 
KP
, 
Croce
 
CM
 (2010). MicroRNA cluster 221–222 and estrogen receptor alpha interactions in breast cancer. J Natl Cancer Inst.

[73] 
Pogribny
 
IP
, 
Filkowski
 
JN
, 
Tryndyak
 
VP
, 
Golubov
 
A
, 
Shpyleva
 
SI
, 
Kovalchuk
 
O
 (2010). Alterations of microRNAs and their targets are associated with acquired resistance of MCF-7 breast cancer cells to cisplatin. Int J Cancer.

[74] 
Lambertini
 
E
, 
Lolli
 
A
, 
Vezzali
 
F
, 
Penolazzi
 
L
, 
Gambari
 
R
, 
Piva
 
R
 (
2012
). Correlation between Slug transcription factor and miR-221 in MDA-MB-231 breast cancer cells. BMC Cancer.

[75] 
Zhao
 
R
, 
Wu
 
J
, 
Jia
 
W
, 
Gong
 
C
, 
Yu
 
F
, 
Ren
 
Z
, 
Chen
 
K
, 
He
 
J
, 
Su
 
F
 (2011). Plasma miR-221 as a predictive biomarker for chemoresistance in breast cancer patients who previously received neoadjuvant chemotherapy. Onkologie.

[76] 
Velu
 
CS
, 
Grimes
 
HL
 (2012). Utilizing antagomiR (antisense microRNA) to knock down microRNA in murine bone marrow cells. Methods Mol Biol.

[77] 
Poltronieri
 
P
, 
D’Urso
 
PI
, 
Mezzolla
 
V
, 
D’Urso
 
OF
 (2013). Potential of anti-cancer therapy based on anti-miR-155 oligonucleotides in glioma and brain tumours. Chem Biol Drug Des.

[78] 
Ma
 
D
, 
Tao
 
X
, 
Gao
 
F
, 
Fan
 
C
, 
Wu
 
D
 (2012). miR-224 functions as an onco-miRNA in hepatocellular carcinoma cells by activating AKT signaling. Oncol Lett.

[79] 
Nielsen
 
PE
, 
Egholm
 
M
, 
Berg
 
RH
, 
Buchardt
 
O
 (1991). Sequence-selective recognition of DNA by strand displacement with a thymine-substituted polyamide. Science.

[80] 
Demidov
 
VV
, 
Frank-Kamenetskii
 
MD
 (2001). Sequence-specific targeting of duplex DNA by peptide nucleic acids via triplex strand invasion. Methods.

[81] 
Gambari
 
R
 (2001). Peptide-nucleic acids (PNAs): a tool for the development of gene expression modifiers. Curr Pharm Des.

[82] 
Karkare
 
S
, 
Bhatnagar
 
D
 (2006). Promising nucleic acid analogs and mimics: characteristic features and applications of PNA, LNA, and morpholino. Appl Microbiol Biotechnol.

[83] 
Nielsen
 
PE
 (2002). Antisense peptide nucleic acids. Curr Opin Mol Ther.

[84] 
Soomets
 
U
, 
Hällbrink
 
M
, 
Langel
 
U
 (1999). Antisense properties of peptide nucleic acids. Front Biosci.

[85] 
Ray
 
A
, 
Nordén
 
B
 (2000). Peptide nucleic acid (PNA): its medical and biotechnical applications and promise for the future. FASEB J.

[86] 
Nielsen
 
PE
 (2001). Targeting double stranded DNA with peptide nucleic acid (PNA). Curr Med Chem.

[87] 
Gambari
 
R
 (2004). Biological activity and delivery of peptide nucleic acids (PNA)-DNA chimeras for transcription factor decoy (TFD) pharmacotherapy. Curr Med Chem.

[88] 
Corradini
 
R
, 
Sforza
 
S
, 
Tedeschi
 
T
, 
Totsingan
 
F
, 
Marchelli
 
R
 (2007). Peptide nucleic acids with a structurally biased backbone: effects of conformational constraints and stereochemistry. Curr Top Med Chem.

[89] 
Sforza
 
S
, 
Tedeschi
 
T
, 
Calabretta
 
A
, 
Corradini
 
R
, 
Camerin
 
C
, 
Tonelli
 
R
, 
Pession
 
A
, 
Marchelli
 
R
 (2010). A peptide nucleic acid embedding a pseudopeptide nuclear localization sequence in the backbone behaves as a peptide mimic. Eur J Org Chem.

[90] 
Sforza
 
S
, 
Corradini
 
R
, 
Ghirardi
 
S
, 
Dossena
 
A
, 
Marchelli
 
R
 (2000). DNA binding of a D-Lysine-based chiral PNA: direction control and mismatch recognition. Eur J Org Chem.

[91] 
Sforza
 
S
, 
Tedeschi
 
T
, 
Corradini
 
R
, 
Marchelli
 
R
 (2007). Induction of helical handedness and DNA binding properties of peptide nucleic acids (PNAs) with two stereogenic centres. Eur J Org Chem.

[92] 
Tedeschi
 
T
, 
Sforza
 
S
, 
Corradini
 
R
, 
Marchelli
 
R
 (2005). Synthesis of new chiral PNAs bearing a dipeptide-mimic monomer with two lysine-derived stereogenic centres. Tetrahedron Lett.

[93] 
Dragulescu-Andrasi
 
A
, 
Zhou
 
P
, 
He
 
G
, 
Ly
 
DH
 (2005). Cell-permeable GPNA with appropriate backbone stereochemistry and spacing binds sequence-specifically to RNA. Chem Commun.

[94] 
Brognara
 
E
, 
Fabbri
 
E
, 
Aimi
 
F
, 
Manicardi
 
A
, 
Bianchi
 
N
, 
Finotti
 
A
, 
Breveglieri
 
G
, 
Borgatti
 
M
, 
Corradini
 
R
, 
Marchelli
 
R
, 
Gambari
 
R
 (2012). Peptide nucleic acids targeting miR-221 modulate p27
^
Kip1
^
expression in breast cancer MDA-MB-231 cells. Int J Oncol.

[95] 
Gambari
 
R
, 
Fabbri
 
E
, 
Borgatti
 
M
, 
Lampronti
 
I
, 
Finotti
 
A
, 
Brognara
 
E
, 
Bianchi
 
N
, 
Manicardi
 
A
, 
Marchelli
 
R
, 
Corradini
 
R
 (2011). Targeting microRNAs involved in human diseases: a novel approach for modification of gene expression and drug development. Biochem Pharmacol.

[96] 
Fabani
 
MM
, 
Gait
 
MJ
 (2008). miR-122 targeting with LNA/2′-O-methyloligonucleotide mixmers, peptide nucleic acids (PNA), and PNA-peptide conjugates. RNA.

[97] 
Fabani
 
MM
, 
Abreu-Goodger
 
C
, 
Williams
 
D
, 
Lyons
 
PA
, 
Torres
 
AG
, 
Smith
 
KGC
 (2010). Efficient inhibition of miR-155 function in vivo by peptide nucleic acids. Nucleic Acids Res.

[98] 
Fabbri
 
E
, 
Manicardi
 
A
, 
Tedeschi
 
T
, 
Sforza
 
S
, 
Bianchi
 
N
, 
Brognara
 
E
, 
Finotti
 
A
, 
Breveglieri
 
G
, 
Borgatti
 
M
, 
Corradini
 
R
, 
Marchelli
 
R
, 
Gambari
 
R
 (2011). Modulation of the biological activity of microRNA-210 with peptide nucleic acids (PNAs). Chem Med Chem.

[99] 
Fabbri
 
E
, 
Brognara
 
E
, 
Borgatti
 
M
, 
Lampronti
 
I
, 
Finotti
 
A
, 
Bianchi
 
N
, 
Sforza
 
S
, 
Tedeschi
 
T
, 
Manicardi
 
A
, 
Marchelli
 
R
, 
Corradini
 
R
, 
Gambari
 
R
 (2011). miRNA therapeutics: delivery and biological activity of peptide nucleic acids targeting miRNAs. Epigenomics.

[100] 
Manicardi
 
A
, 
Fabbri
 
E
, 
Tedeschi
 
T
, 
Sforza
 
S
, 
Bianchi
 
N
, 
Brognara
 
E
, 
Gambari
 
R
, 
Marchelli
 
R
, 
Corradini
 
R
 Cellular uptakes, biostabilities and anti-miR-210 activities of chiral arginine-PNAs in leukaemic K562 cells. Chembiochem.

[101] 
Yan
 
LX
, 
Wu
 
QN
, 
Zhang
 
Y
, 
Li
 
YY
, 
Liao
 
DZ
, 
Hou
 
JH
, 
Fu
 
J
, 
Zeng
 
MS
, 
Yun
 
JP
, 
Wu
 
QL
, 
Zeng
 
YX
, 
Shao
 
JY
 (2011). Knockdown of miR-21 in human breast cancer cell lines inhibits proliferation, in vitro migration and in vivo tumor growth. Breast Cancer Res.

